# Examining the Relationship Between Time Spent on Social Media Platforms and Body Image Concerns

**DOI:** 10.1002/erv.70030

**Published:** 2025-09-09

**Authors:** Kavya Malhotra, Stephanie Miles, Eric J. Tan, Andrea Phillipou

**Affiliations:** ^1^ Department of Psychological Sciences Swinburne University of Technology Melbourne Australia; ^2^ Orygen Melbourne Australia; ^3^ Centre for Youth Mental Health The University of Melbourne Melbourne Australia; ^4^ Memory Aging & Cognition Centre National University Health System Singapore Singapore; ^5^ Department of Pharmacology Yong Loo Lin School of Medicine National University of Singapore Queenstown Singapore; ^6^ Centre for Mental Health and Brain Sciences Swinburne University of Technology Melbourne Australia; ^7^ Orygen Specialist Program Royal Melbourne Hospital Melbourne Australia; ^8^ Department of Mental Health St Vincent's Hospital Melbourne Australia; ^9^ Department of Mental Health Austin Health Melbourne Australia

**Keywords:** body dissatisfaction, body image, eating disorder, social media, TikTok

## Abstract

**Objective:**

Research has suggested that a relationship may exist between frequent use of social networking sites (SNSs) and body dissatisfaction; however, there is a lack of research around newer SNS platforms with larger visual imprints, such as TikTok and Snapchat. The aim of the present study was to investigate the relationship between time spent on different SNSs and body dissatisfaction.

**Methods:**

An online survey was completed by 199 participants.

**Results:**

A hierarchical linear regression did not reveal a significant relationship between overall time spent on SNSs and body dissatisfaction. A stepwise regression of time spent on individual SNSs revealed that only time spent on TikTok was significantly associated with body dissatisfaction.

**Conclusion:**

The findings suggest that the type of SNSs used, and not the overall time spent on SNSs, may be driving relationships with levels of body dissatisfaction. Further research is required to clarify how different SNS types relate to body image.

## Highlights

- No relationship between overall time spent on social media and body dissatisfaction
- Significant association between time spent on TikTok and body dissatisfaction
- Specific social media use may be related to body image

## 1 Introduction

Body dissatisfaction is high across the age spectrum for males and females (Quittkat et al. 2019) and has been found to remain relatively stable in women as they age (Slevec and Tiggemann 2011). Understanding the potential contributors to body dissatisfaction is thus of critical importance. Perceived pressure from traditional media sources (television, magazines, print advertising) is notable for its association with appearance internalization and appearance comparisons, consequently being reported to result in body dissatisfaction (Munsch et al. 2021; Rodgers et al. 2015); however, there has been a steep decline in magazine readership and television viewing, with internet use increasing by approximately 50% each year since 1990 and social media becoming increasingly popular (Holland and Tiggemann 2016). Social media, or social networking sites (SNSs), are defined as virtual platforms that permit users to share, create and consume content within their networks (Pittman and Reich 2016).

Presently, the popularity of SNSs is far outpacing that of traditional media (Guttmann 2023). Empirical evidence has suggested that certain aspects of SNS use may be harmful to psychological wellbeing, especially in relation to body image (Blackford 2020; Holland and Tiggemann 2016). While research has only recently started exploring the nuanced effects of SNSs on body image, increased time spent on SNSs has been linked to a range of behaviours associated with increased body dissatisfaction such as body surveillance (a preoccupation with monitoring one's attractiveness), appearance investment, and increased weight and shape concerns (Butkowski et al. 2019; Manago et al. 2015; Strubel and Petrie 2017; Wick and Keel 2020). SNS use (e.g. excessive time spent, appearance‐based use, using filters, online feedback) has been shown to be associated with negative body image in adolescents (De Vries et al. 2016; Jarman, Marques, McLean, Slater, and Paxton 2021b), young adults (Engeln et al. 2020; Fardouly and Vartanian 2015), older women (Hockey et al. 2021; Nagl et al. 2021), and men (Piatkowski et al. 2021; Sumter et al. 2022).

The real‐time and personalized aspect of SNSs, exposure to different sources of influence and algorithmising effects (wherein users encounter content based on trends, relevancy, and past use), differentiate it from traditional media, with potentially differing outcomes for body image. The visual nature of SNSs encourages a focus on physical appearance which is also influenced by its quantifiability (likes, comments, followers). This creates potential for peer feedback and approval (often based on appearance in photos), which has consequently been shown to negatively affect body image and shape appearance ideals (Chua and Chang 2016; Nesi et al. 2018). Some studies (e.g. Fardouly et al. 2017; Roberts et al. 2022) have reported that women compared their appearance to others on SNSs more than any other platform (e.g. magazines, television), and the effects of these online comparisons are significantly more detrimental to body image than those on traditional media.

One of the most significant differences between how SNSs and traditional media may affect body image is that SNSs not only depict images of celebrities and models like traditional media, but also peers, who can have particularly high influence upon body image (Hogue and Mills 2019; Holland and Tiggemann 2016). As all targets for comparison (close/distant peers, celebrities etc.) appear on the same platform, users are exposed to and interact with different sources of influence, which may encourage the internalization of body image ideals in a more profound way than these influences in isolation (Meier and Gray 2014; Slattery 2013). Additionally, users are exposed to influence from multiple appearance messages such as blogs, stories, digitally altered photos, comments/likes, videos etc. across different platforms (Santarossa and Woodruff 2017). Thus, communication and social interaction on SNSs consists of an amalgamation of mass media, personal commentaries, status updates/stories, likes and comments on content, and personal messaging. These allow an individual to connect with many people instantaneously, thus creating an interpersonal communication with higher potential for social influences.

Many studies group SNS platforms together, however, due to differences in their function, use and content, they potentially affect body image differently. While Facebook dominates the existing literature (Hummel and Smith 2015; Kim and Chock 2015), research has suggested that ‘appearance‐focused’ SNS platforms may specifically contribute to body image concerns (Meier and Gray 2014; Tiggemann and Anderberg 2020). A meta‐analysis by Mingoia et al. (2017) found a stronger relationship between body dissatisfaction and exposure to appearance‐related content, as opposed to general exposure. Highly visual and appearance‐focused SNS platforms like Instagram, Snapchat and TikTok are those that focus on user‐generated appearance‐focused content and often promote unattainable beauty standards, potentially increasing awareness of contemporary beauty standards leading to associated pressures to conform to these (Maes and Vandenbosch 2022; Marengo et al. 2018). Certain features of these platforms such as digital manipulation, digital filters, and posting/consuming appearance‐related content have implications for body dissatisfaction.

Instagram users have been shown to report higher body surveillance (R. Cohen et al. 2017) and lower body satisfaction (Tiggemann and Anderberg 2020). Studies have shown that following appearance‐focused Instagram accounts, engaging in photo‐based activities, witnessing, and responding to appearance‐ideals, has also been linked to increased body dissatisfaction (Baker et al. 2019; R. Cohen et al. 2017; McLean et al. 2015). Engeln et al. (2020) further reported that women browsing Instagram made more appearance comparisons, thought more about their appearance, and showed decreased body satisfaction and decreased positive affect, compared to using only partly appearance‐focussed platforms such as Facebook. Using Snapchat lenses and filters, engagement in selfies (photos of the self) until reaching a satisfactory one to post on SNSs, and engaging in digital modification, have also been associated with body dissatisfaction for both content creators and consumers (Burnell et al. 2022; Kleemans et al. 2018; Tiggemann and Anderberg 2020; Wick and Keel 2020).

Overall, time spent on more appearance‐based platforms such as Instagram and Snapchat had a greater effect on body dissatisfaction than less appearance‐focused platforms such as Facebook (Marengo et al. 2018). Presently, research with newer appearance‐focussed platforms (i.e., Snapchat, TikTok, Instagram) is just emerging, even though their current use exceeds older SNS platforms such as Facebook (Guttmann 2023). Emerging studies have found positive relationships between TikTok (Mink and Szymanski 2022) and Snapchat use (Marengo et al. 2018) and body dissatisfaction. These newer platforms focus highly on sharing user‐generated visual content such as photos and short videos that are modified using filters (Burnell et al. 2022; Marengo et al. 2018), and their impacts thus need to be further investigated.

Although much of the literature has been focused on adolescents and young adults, social media is used across the age spectrum and body dissatisfaction is also present across the lifespan. Therefore, it is important to understand the potential influence across adulthood. The aim of this study was to investigate the relationship between SNS use and body image concerns in adults, and explore the specific contributions of individual SNS platforms to body dissatisfaction. Specifically, it was hypothesized that increased time spent on social media would be positively related to higher levels of body dissatisfaction. Given the limited data available on the use of different social media platforms, how different SNS platforms (Facebook, Instagram, Snapchat, TikTok and Twitter) contributed to levels of body dissatisfaction was also explored.

## 2 Method

This quantitative empirical study employed a cross‐sectional design via an online Qualtrics survey. The study was approved by the Swinburne University Human Research Ethics Committee.

### 2.1 Participants

Two hundred and seventy participants aged 18 years and over responded to the survey between May 2022 and July 2022. Participants were recruited through the Research Experience Program Website (REP) program at Swinburne University, social media posts, and participant registries held at Swinburne University. Participants residing in Australia were offered the opportunity to enter a draw to win one of eight $25 vouchers, and REP students were offered course credit for completing the survey. Participants have given consent for their data to be used in the research.

Prior to data analysis, data were checked for accuracy of data entry and missing values. Sixty‐four cases were removed listwise as they did not complete the Eating Disorders Examination Questionnaire. One typographic error was cleaned from the data. Six participants were removed as they failed to pass two out of three attention check questions. Following data cleaning, a final sample of 199 adult participants (24 males, 175 females) aged 18–82 years (*M* = 29.9, SD = 10.55), were included in the analyses. Forty‐eight participants (29.1%) of the final sample reported a current or past eating disorder. The sample was 78.4% Caucasian (*n* = 156), 14.1% (*n* = 28) Asian, 1.5% (*n* = 3) Aboriginal or Torres Strait Islander, 1% (*n* = 1) African, and 6% (*n* = 12) identified as another race. The mean years of education for the whole sample was 16.59 (SD = 2.57), with 77.9% (*n* = 156) of respondents born in Australia.

### 2.2 Measures

This study is part of a larger project that investigated a broad range of influences on body image; only the measures related to the current study are described here. Participants completed a demographic questionnaire and were asked to indicate current/past diagnosis of mental illness. The outcome variable, body dissatisfaction, was operationalized using the shape and weight concern subscales of the EDE‐Q (Fairburn and Beglin 2008), The EDE‐Q has excellent internal consistency (Cronbach's alpha was 0.84, 0.93, 0.89 and 0.78 for the Restraint, Shape Concern, Weight Concern and Eating Concern subscales respectively) and 2‐week test‐retest reliability for all subscales (*p* < 0.001 (Luce and Crowther 1999)). For this study, the shape and weight concern subscales of the EDE‐Q were summed then divided by the number of items forming the subscales to obtain a body dissatisfaction score. Higher scores indicated more severe body dissatisfaction.

SNS use was operationalized as the average number of minutes per day (over the past week) participants reported spending on five specified SNS platforms, that is Facebook, Instagram, Snapchat, Twitter and TikTok. This variable was adapted from previous studies that measured time spent on SNSs (Santarossa and Woodruff 2017). A summed score for time spent across all platforms and the time spent on each social media platform separately were calculated. Participants who did not use specific platforms were coded as spending 0 min on the platform.

### 2.3 Statistical Analysis

The primary hypothesis was examined using a hierarchical linear regression with body dissatisfaction as the outcome variable and overall time spent across five SNS platforms as the independent variable. Relevant demographic variables, that is, age and sex, were included as covariates in Block 1 to account for their potential influence on body dissatisfaction. In Block 2, the number of platforms was also included to account for breadth of SNS use. The summed time spent across all platforms was added in Block 3. Utilizing a medium effect size and 80% power, a minimum of 55 participants was required to address our primary hypothesis.

To explore the contribution of time spent on each specific SNS on body dissatisfaction, a stepwise linear regression was performed. Demographic variables of age and sex were included as covariates in Block 1, and time spent on each respective platform included in Block 2. Data were analysed using IBM Statistical Package for Social Sciences (SPSS) 28.0.1.

## 3 Results

A summary of body dissatisfaction scores, as well as social media use, are reported in Table 1.

**TABLE 1:** Body dissatisfaction (DV) and social media use.

	*n* (%)	*M* (SD)
Variable		
Body dissatisfaction		3.10 (1.81)
Sum time spent (in minutes)		236.62 (292.55)
Number of platforms used		2.79 (1.25)
SNS use, *n* (%)/Number of minutes per week, *M (*SD)		
Facebook	174 (87.4%)	66.77 (98.2)
Instagram	165 (82.9%)	79.95 (108.48)
Snapchat	94 (47.2%)	27.38 (75.75)
TikTok	82 (41.2%)	55.06 (135.3)
Twitter	41 (20.6%)	7.43 (33.15)

*Note:* Body dissatisfaction was obtained by summing the shape and weight concern subscales of the Eating Disorders Examination Questionnaire and divided by the number of items.

Results for the regression analyses as reported in Tables 2 and 3. The correlational matrix is presented in Supporting Information S1: Table S1. For the primary hypothesis investigating the sum time spent on body dissatisfaction, Block 1 was significant, *R* ^*2*^ = 0.087, *F* (2,196) = 9.37, *p* < 0.001, with age and sex accounting for a combined 8.7% of the variance in body dissatisfaction. The addition of number of platforms in Block 2 did not result in a significant *R* ^*2*^ change (*R* ^*2*^ *=* 0.10, *F* (3.195) = 7.24, *p* = 0.096), accounting for only an additional 1.3% variance on top of that of Block 1. The sum time spent across platforms included in Block 3 accounted for an additional 0.6% variance on top of the previous models, *R* ^*2*^ = 0.106, *F* (4,194) = 5.76, *p* = 0.255, and was also not significant.

**TABLE 2:** Regression statistics for sum of time spent on social media and body dissatisfaction.

Predictor	Standardised *β*	*t*	95% CI Intervals		
			Upper limit	Lower limit	Sig.
Model 1					
Age	−0.05	−0.77	−0.03	0.01	0.44
Sex	0.29	4.23	0.85	2.34	< 0.001
Model 2					
Age	0.01	0.10	−0.02	0.03	0.92
Sex	0.29	4.28	0.87	2.35	< 0.001
Number of platforms	0.13	1.67	−0.03	0.40	0.10
Model 3					
Age	0.02	0.22	−0.02	0.03	0.83
Sex	0.30	4.38	0.91	2.40	< 0.001
Number of platforms	0.10	1.27	−0.08	0.374	0.20
Sum time spent	0.08	1.14	0.00	0.001	0.25

Abbreviations: CI = Confidence Intervals, Sum Time Spent = sum of time spent across Facebook, Instagram, Snapchat, TikTok, Twitter.

**TABLE 3:** Regression statistics for the time spent on different social media platforms on body dissatisfaction.

Predictor	Standardised *β*	t	95% CI Intervals		Sig.
			Upper limit	Lower limit	
Model 1					
Age	−0.05	−0.77	−0.03	0.014	0.44
Sex	0.29	4.23	0.85	2.34	< 0.001
Model 2					
Age	0.01	−0.17	−0.02	0.03	0.92
Sex	0.29	4.55	0.87	2.35	< 0.001
Facebook	−0.05	−0.68	—	—	0.50
Instagram	0.03	0.67	—	—	0.67
Snapchat	0.03	0.34	—	—	0.73
TikTok	0.15	2.05	0.00	0.004	0.04
Twitter	0.02	0.27		—	0.79

Abbreviation: CI = Confidence Intervals.

For the secondary hypothesis (Table 3), which explored the relationship between time spent on different social media platforms and body dissatisfaction, only time spent on TikTok was significant, *R* ^*2*^ = 0.093, *F* (1,195) = 7.75, *p* = 0.042, explaining an additional 1.9% of variance on top of age and sex. Time spent on Facebook, Instagram, Twitter and Snapchat did not significantly contribute to the model.

## 4 Discussion

The primary hypothesis that overall time spent on SNSs would have a positive relationship with body dissatisfaction in adults was not supported. The secondary exploratory hypothesis investigating whether time spent on specific SNS platforms differed in their relationship to levels of body dissatisfaction indicated that only time spent using TikTok significantly contributed to body dissatisfaction.

### 4.1 Overall Time Spent on SNSs and Body Dissatisfaction

Our findings corroborate a handful of studies that operationalized SNS use as time spent on SNSs, and similarly reported no relationship between time spent on SNSs and body dissatisfaction (J. Cohen 2013; Fardouly and Vartanian 2015; Kim and Chock 2015). Simultaneously, the findings also contrast with other previous studies that found significant positive relationships between SNS use (measured as time spent) and indicators of body dissatisfaction such as internalization of the thin‐ideal, increased body shame, body surveillance, body comparisons and reduced body esteem (Marengo et al. 2018; Tiggemann and Slater 2013). A potential reason for the discrepancy may be the use of an adult sample in the current study, with many previous studies finding a significant relationship between social media use and body dissatisfaction investigating adolescents (e.g. (De Vries et al. 2016; Holland and Tiggemann 2016; Marengo et al. 2018)). Notably, young people are particularly vulnerable to negative body image due to developmental changes and socio‐cultural and peer related pressures (Saiphoo and Vahedi 2019; Voelker et al. 2015).

### 4.2 Operationalization of SNS Use

Some studies have concluded that operationalizing SNS use as time spent may not necessarily capture the nuances of SNS use (R. Cohen et al. 2017; Jarman et al. 2021a, 2021b). The concept of SNSs is not unitary, with marked differences across SNS platforms such as stories, reels, likes, captions, trends, reactions, hashtags, filters etc, which the present study did not examine. The current findings suggest that overall time spent on SNSs may not be related to body dissatisfaction, although time spent on individual platforms may be a more reflective measure. Overall, future investigations should consider focussing on other SNS metrics as well.

### 4.3 Time Spent Between Different SNSs and Body Dissatisfaction

The finding that only time spent on TikTok, and not other SNS platforms, was associated with body dissatisfaction is aligned with evidence that time spent on TikTok was indirectly related to body dissatisfaction through body surveillance and upward appearance comparisons (Mink and Szymanski 2022). The current findings contrast, however, with a recent longitudinal investigation that reported that time spent on TikTok was not related to body image self‐discrepancy or internalization of beauty ideals over time (Maes and Vandenbosch 2022). The discrepancy may be attributed to how the current study investigated shape and weight concern specifically, or it may be possible that appearance‐based platforms only contribute to body dissatisfaction in the short‐term. Overall, existing findings on how TikTok use relates to body dissatisfaction are inconsistent. Thus, while the current study supports a direct link between body dissatisfaction and TikTok use, the prevailing inconsistencies in the field support the need for further research into the mechanisms underlying this relationship.

A potential explanation for the current findings may lie in the nature of TikTok use. Affirmation from others and social pressure are some of the main motivational reasons users engage in active TikTok use (Falgoust et al. 2022; Roth et al. 2021), and considering the prevalence of peer influence in maintaining body image concerns (Hogue and Mills 2019), it may be expected that increased TikTok use would be related to higher body dissatisfaction. Due to its algorithmising nature, and viral trends that revolve around and fixate on appearance, TikTok can have a negative impact upon both mental health and body image, wherein a user encounters an array of personalized and customized content based on previous engagement and use (Bhandari and Bimo 2020; Liu 2021). Further, unlike other platforms, TikTok users do not need to follow accounts to passively access their content and can thus be subjected to an endless array of consumable content (Liu 2021). Thus, one may merely signal preference for pro‐eating disorder content, or idealized and photoshopped bodies and then would passively be subjected to this content online.

### 4.4 Limitations

A limitation of the study is that the protocol and planned analyses were not pre‐registered. Regarding methodological limitations, while the current study controlled for demographic covariates such as sex and age which can affect the relationship between SNS use and body dissatisfaction (Faelens et al. 2021; Marques et al. 2022), it did not address individual differences such as appearance‐investment, internalization and appearance comparison tendencies that have been shown to mediate relationships between SNS use and body dissatisfaction (Betz et al. 2019; R. Cohen et al. 2018; Fardouly et al. 2017; Strubel and Petrie 2017). Furthermore, the cross‐sectional nature of this study limits direction of causality. While SNS use may affect body image concerns, the converse is also plausible. For example, it is possible that those with lower body satisfaction may consciously seek out TikTok to view digitally altered images and use filters and pursue appearance‐related feedback. Further longitudinal and experimental studies can help establish conclusions about causality. In addition, about 1/3 of the study sample were individuals with a current/past diagnosis of an eating disorder. Given how eating disorder symptomology can arise from body image concerns (Fardouly et al. 2017), conducting studies with larger samples of participants with diagnosed eating disorders could ascertain whether there are differences or similarities in the relationship between SNS use and body dissatisfaction in individuals with eating disorders.

There was an uneven distribution between males and females in the sample which could have affected the results and limits the generalizability of the current study. Further research investigating the relationship between time spent on social media and body dissatisfaction in specific genders would be beneficial for future research, as would be the specific investigation of gender diverse populations given the body image distress often experienced in this group (Jones et al. 2018; Milano et al. 2020). In addition, future research would benefit from exploring whether differences exist in individuals who use all SNS platforms to identify unique or shared variance, as well as whether body mass index influences results.

## 5 Conclusion

As much of the literature is focused on adolescents and young adults, a key strength of the current study is the investigation of SNS use and its relationship to body dissatisfaction among adults. Overall, the current findings suggest that time on specific SNSs may contribute to negative body image more so than overall time spent on SNSs. Further research specifically examining the nuances of SNS use such as utilizing filters, posting photos, engaging in likes and comments, would be beneficial as time spent on SNSs may not capture the content, activities, and behaviour exhibited across platforms and how it may be related to body image concerns. The present study also highlights the need to further investigate novel SNS platforms such as TikTok due to their highly appearance‐focused nature of content and use, and explore both direct and indirect relations between TikTok use and body dissatisfaction. Longitudinal and experiential studies, investigating individual differences such as appearance investment, appearance comparisons and internalisations would also be beneficial. Understanding these relationships will potentially help inform SNS literacy programs and tools to combat the negative effects of SNS use on body image concerns.

## Ethics Statement

The study was approved by Swinburne University Human Ethics Committee (reference number: 20226418‐9819).

## Consent

All participants provided informed consent to participate.

## Conflicts of Interest

The authors declare no conflicts of interest.

## Supporting information


**Table S1:** Pearson’s correlation analyses between body dissatisfaction and other regression variables.

## Data Availability

Data can be requested from the corresponding author upon reasonable request.
