# Supplementary material for: Examining the Relationship Between Time Spent on Social Media Platforms and Body Image Concerns
Source: Eur Eat Disord Rev. 2025 Sep 9;34(2):313–20. doi: 10.1002/erv.70030 (PMC12862517; doi:10.1002/erv.70030)
Supplement: Supplementary file 1 — Table S1: Pearson’s correlation analyses between body dissatisfaction and other regression variables. [file ERV-34-313-s001.docx]

Supplementary Material

Supplementary Table 1: Pearson’s correlation analyses between body dissatisfaction and other regression variables

| *Variable* | 1 | 2 | 3 | 4 | 5 | 6 | 7 | 8 |
| --- | --- | --- | --- | --- | --- | --- | --- | --- |
| 1.Body  Dissatisfaction | - | -.06 | .29** | .12 | .09 | - | - | - |
| 2. Age | -.06 | - | -.04 | -.46 | -.25 | - | - | - |
| 3**.** Sex***** | .29** | -.04 | - | .0010 | -.09 | - | - | - |
| 4. Number of  Platforms Used | .12 | -.46 | .0010 | - | .37 | - | - | - |
| 5. Sum Time  Spent | .09 | -.25 | -.09 | .37 | - | - | - | - |
| 6. Facebook | -.06 | .03 | -.13 | - | .51** | .04 | .19 | -.04 |
| 7. Instagram | .09 | -.19 | .03 | .51* | - | .15 | .34** | .09 |
| 8. Snapchat | .01 | -.23** | .03 | .04 | .15 | - | .44** | .01 |
| 9. TikTok | .10 | -.27** | -.15 | .19 | .34** | .44** | - | .09 |
| 10. Twitter | .03 | -.11 | -.01 | -.04 | .09 | .01 | .09 | - |

*Note*: *Spearman’s correlation for sex as it is categorical, ** *p*<.01
